# Pellino1 promotes chronic inflammatory skin disease via keratinocyte hyperproliferation and induction of the T helper 17 response

**DOI:** 10.1038/s12276-020-00489-4

**Published:** 2020-09-01

**Authors:** Suhyeon Kim, Si-Yeon Lee, Seoyoon Bae, Jin-Kwan Lee, Kyungrim Hwang, Heounjeong Go, Chang-Woo Lee

**Affiliations:** 1 Department of Molecular Cell Biology, Samsung Medical Center, Sungkyunkwan University School of Medicine, Suwon, 16419 Republic of Korea; 2 Department of Health Sciences and Technology, SAIHST, Sungkyunkwan University, Seoul, 06351 Republic of Korea; 3 Department of Pathology, University of Ulsan College of Medicine, Asan Medical Center, Seoul, 05505 Republic of Korea

**Keywords:** Chronic inflammation, Immunoproliferative disorders

## Abstract

Psoriasis is one of the most common immune-mediated chronic inflammatory skin diseases. However, little is known about the molecular mechanism underlying the immunological circuits that maintain innate and adaptive immune responses in established psoriasis. In this study, we found that the Pellino1 (Peli1) ubiquitin E3 ligase is activated by innate pattern-recognition receptors (PRRs), such as Toll-like receptors (TLRs), and is highly upregulated in human psoriatic skin lesions and murine psoriasis-like models. Increased Peli1 expression is strongly correlated with the immunopathogenesis of psoriasis by activating hyperproliferation of keratinocytes in the S and G2/M phases of the cell cycle and promoting chronic skin inflammation. Furthermore, Peli1-induced psoriasis-like lesions showed significant changes in the expression levels of several T helper 17 (Th17)-related cytokines, such as IL-17a, IL-21, IL-22, IL-23, and IL-24, indicating that overexpression of Peli1 resulted in the sequential engagement of the Th17 cell response. However, the overexpression of Peli1 in T cells was insufficient to trigger psoriasis, while T cells were indispensable for disease manifestation. In summary, our findings demonstrate that Peli1 is a critical cell cycle activator of innate immunity, which subsequently links Th17 cell immune responses to the psoriatic microenvironment.

## Psoriasis: Regulatory protein linked to chronic inflammation

An immune-regulating protein that mediates chronic inflammation in the skin offers a new therapeutic target for the autoimmune disorder psoriasis. A research team from South Korea led by Chang-Woo Lee from Sungkyunkwan University School of Medicine in Suwon and Heounjeong Go from the University of Ulsan College of Medicine in Seoul have discovered that Pellino1, a protein known to modulate immune responses to pathogens, is also found in abundance in the skin lesions of people with psoriasis. Using mouse models, the researchers showed how Pellino1 induces the proliferation of certain skin cells and triggers an inflammatory state through the activation of small proteins and immune cells normally involved in defense against infection. Targeting strategy that inactivate Pellino1 could help blunt the inflammatory signaling in the skin that drives the development of psoriatic lesions.

## Introduction

The development of psoriasis requires cross-talk between innate immunity and adaptive immune responses. Dysregulation of the skin inflammatory signaling pathway results in keratinocyte hyperproliferation and is one of the key pathogenic mechanisms underlying the development of psoriasis^1^. In psoriatic skin lesions, both αβ and γδ T cells synthesize IL-17, while activated Dendritic cells (DCs) secrete IL-23 and IL-12. They stimulate three populations of resident T cells: Th17, Th22, and Th1 cells^2,3^. IL-23 activates Th17 cells to release IL-17A and IL-17F, which induce keratinocyte hyperactivation. DCs also play an essential role in the conversion of nonlesional to lesional psoriatic skin in a transplantation model^4^. Thus, T cell-derived and DC-derived cytokines act on epidermal keratinocytes as proximal inducers of immunogenic circuits in psoriasis. Upon activation, skin epidermal cells release an abundance of cytokines, chemokines, and other inflammatory mediators, including CXCL8, monocyte chemotactic protein-1/CCL2, CXCL1-3, and CCL20^5–7^. However, selective activation of immune cells alone is not sufficient for the initiation of psoriasis.

The initial recognition of pathogens is mediated by innate pattern-recognition receptors (PRRs) that detect conserved microbial structures of invading pathogens or endogenous molecules released from damaged cells^8,9^. PRRs are expressed by many cell types, including nonimmune cells^8,10^. In some cases, they recognize pathogen-associated molecular patterns (PAMPs) and damage-associated molecular patterns (DAMPs), similar to Toll-like receptors (TLRs)^9,11^. Such TLR-mediated recognition of PAMPs and DAMPs triggers the expression of several adapter proteins and downstream kinases, leading to the induction of key proinflammatory mediators, which results in the activation of both the innate immune response mediated via enhanced expression of cell survival proteins and proinflammatory cytokines as well as the adaptive immune response mediated via maturation of DCs and antigen presentation. Accumulating evidence has also demonstrated that TLRs are widely expressed in the autoimmune microenvironment, and the activation of TLRs boosts the autoimmune response^9–12^.

Pellino1 (Peli1) is an E3 ubiquitin ligase. It is the regulator of PRR signaling and innate immune responses^8,13,14^. Recent studies have unveiled a critical role of Peli1 in activating TLR and/or T cell receptor (TCR) signaling-mediated proinflammatory gene expression^15–18^. Notably, Peli1 expression is highly suppressed under normal or nonpathological conditions. In contrast, pathogenic conditions promote Peli1 expression. For instance, Peli1 expression is upregulated in patients with neutrophilic asthma^19^ and those diagnosed with diffuse large B cell lymphoma^13^. Moreover, Peli1 expression is associated with poor outcome in B cell lymphomas^13^, suggesting that constitutive expression of Peli1 contributes to the development of chronic inflammatory immune diseases and tumors. Interestingly, Peli1 expression is activated in response to various receptor-mediated signaling events, such as those associated with TLRs, TCRs, and BCRs^16,17,20^. Thus, aberrant regulation of these receptor-mediated signaling pathways can induce an imbalance in the activation or expression of Peli1, which triggers forced signal cascades, ultimately contributing to the development of diseases, such as autoimmune disease. The objective of this study, which is based on gain-of-function and loss-of-function approaches, is to determine whether Peli1 contributes to the establishment of an immunopathological circuit by linking innate immunity to adaptive immune responses.

## Materials and methods

### Animal studies

To generate doxycycline-inducible human Peli1-transgenic mice (rtTA-Peli1), we crossed pTRE TetO Myc-Peli1 transgenic mice with R26-M2rtTA mice (B6.Cg-Gt(ROSA)26Sor^tm1(rtTA*M2)Jae^/J, Jackson Laboratory). These animals were maintained under specific pathogen-free conditions. All experiments were performed with mice homozygous for both the R26-M2rtTA transgene and the human Peli1 transgene. Genotyping was performed with Ready-2X-GO polymerase mixture (HelixAmp^TM^) using genomic DNA isolated from tail biopsy samples. To induce the expression of the human Peli1 transgene, 4-week-old mice were provided drinking water containing 2 mg/mL doxycycline (Sigma) and 5% sucrose (Sigma) for the indicated time period. Water was protected from light and refreshed every three days.

All animal experiments were conducted in accordance with guidelines of the Institutional Animal Care and Use Committee (IACUC) of Sungkyunkwan University School of Medicine (SUSM). SUSM is accredited by the Association for Assessment and Accreditation of Laboratory Animal Care International (AAALAC International). It abides by the Institute of Laboratory Animal Resources (ILAR) guidelines.

### IMQ-induced psoriasis model and DNCB-induced atopic dermatitis model

Mice (C57BL/6) at 6 weeks of age were shaved dorsally and treated with a topical application of 62.5 mg of imiquimod (IMQ) daily using a commercially available cream (Aldara; 3 M Pharmaceuticals) for 6–8 consecutive days. Atopic dermatitis lesions were induced in 6-week-old male BALB/c mice using dinitrochlorobenzene (DNCB). Briefly, the dorsal hair was removed, and then 100 μL of 1% (w/v) DNCB solution (dissolved in a 3:1 [v:v] mixture of olive oil and acetone) was applied to the back skin for sensitization. Five days after dorsal hair removal, 0.2% DNCB (150 μL) was used to challenge the dorsal skin three times per week for three weeks.

### Severity scoring of skin inflammation

To score the severity of psoriasiform inflammation, an objective scoring system was developed based on the clinical psoriasis area and severity index (PASI). Erythema and scaling were scored on a scale ranging from 0 to 5 (0. none; 1. slight; 2. moderate; 3. marked; 4. severe; and 5. very severe). Acanthosis was scored on a scale from 0 to 5 based on thickening of the skin epidermis [0. 5–10 μm (normal); 1. 10–20 μm; 2. 20–30 μm; 3. 30–60 μm; 4. 60–90 μm; and 5. >90 μm]. Erythema, scaling, and acanthosis were scored independently. The average score (erythema combined with scaling and thickening) was used to measure the severity of psoriasis (scale: 0–5).

### Flow cytometry

Lymphocytes were obtained from the blood, spleen, and lymph nodes. Erythrocytes were lysed. Single-cell suspensions were prepared in phosphate-buffered saline (PBS) and stained with fluorophore-conjugated antibodies. All antibodies used in this study are listed in Supplementary Table 1. For intracellular staining, single-cell suspensions were obtained from skin draining lymph nodes and cultured for 5 h with PMA (50 ng/mL) plus ionomycin (500 ng/mL). Brefeldin A (Thermo Fisher Scientific) was added during the final 4 h of incubation. After stimulation, the cells were washed with PBS and fixed with intracellular fixation buffer (Thermo Fisher Scientific) followed by cell permeabilization with permeabilization buffer (Thermo Fisher Scientific). The cells were stained with fluorescently conjugated anticytokine antibodies. Data were obtained using a Canto II flow cytometer (BD Biosciences) and analyzed with FlowJo software (FlowJo, LLC).

### Bone marrow transplantation (BMT)

A schematic illustration of bone marrow chimera experiments is shown in Fig. 3. Briefly, male recipient mice were exposed to 10 Gy of total body irradiation. Male donor mice were euthanized under CO_2_ asphyxiation. The femur and tibia were then isolated from donor mice. Bone marrow cells were flushed out of the femur and tibia bones into sterile RPMI supplemented with 10% FBS. Following isolation, red blood cells were lysed using RBC lysis buffer (Thermo Fisher Scientific) until the cell pellet was void of red color. Next, the cell suspension was strained through a 70-μm cell strainer. Live cell counts were obtained using a hemocytometer after trypan blue staining. Cells were resuspended in Hanks Balanced Salt Solution (HBSS) at a concentration of 25 × 10^6^ cells/mL and stored on ice until transplantation (usually less than 1 h). Approximately 4–5 h following irradiation, 200 μL of isolated bone marrow cells were transplanted into irradiated mice via tail vein injection. Following transplantation, mice were fed normal food and acidified antibiotic water for 14 days for recovery. Recipient mice were bled at two weeks after BMT. Cells were then stained with antiCD45.1 and antiCD45.2 antibodies to determine the engraftment efficiency of BMT.

### Quantitative real-time PCR (qRT-PCR) and immunoblotting

We performed qRT-PCR as previously described^13^ using gene-specific primer sets (Supplementary Table 2) purchased from QIAGEN. Relative gene expression (in triplicate) was assessed after normalization against the expression of glyceraldehyde 3-phosphate dehydrogenase (GAPDH) as a reference gene. All antibodies used in for immunoblotting analyses are listed in Supplementary Table 3.

### Patients and immunohistochemistry

The retrospective patient cohort comprised 156 patients with psoriasis using formalin-fixed paraffin-embedded (FFPE) tissues derived from the primary biopsies of their skin lesion samples at Asan Medical Center from 2010 to 2012. Among them, a total of 18 patients diagnosed with psoriasis via clinical and histological evaluation were consecutively included in this study. Additionally, five normal breast skin tissues obtained from reduction surgery served as controls. Histopathological features of each case were reviewed by a dermatopathologist. Representative histological findings of psoriasis were confirmed for all included cases.

To evaluate Peli1 expression in human psoriatic lesions, immunohistochemical staining (IHC) was performed with an antimouse Pellino1 polyclonal antibody using a BenchMark XT automated system (Ventana Medical Systems) according to the manufacturer’s protocol. Expression of Peli1 was assessed semiquantitatively using the image scan program at each histological site of the skin based on staining intensity and subcellular localization: 1. histological sites: corneal, spinous, and basal layers of epidermis, inflammatory cells, and endothelial cells; 2. staining intensity: 0, no staining; 1+, weak; 2+, moderate; and 3+, strong; and 3. subcellular localization: cytoplasmic and nuclear. This experiment was approved by the Asan Medical Center Institutional Review Board (approval number: 2016-0112).

### Histopathological analysis

Paraffin or frozen tissue blocks of mouse skin biopsies were prepared using routine methods and sectioned to obtain consecutive levels of thickness, followed by staining with hematoxylin and eosin. For immunohistochemical analyses of tissues derived from mice, the sections were deparaffinized, treated with citrate buffer for antigen retrieval, stained with antibodies, and then incubated with an avidin-biotin-horseradish peroxidase complex (Vectastain Elite ABC kit; Vector Laboratories). Finally, peroxidase activity was visualized using a 3,3′-diaminobenzidine substrate kit (Vector Laboratories). Tissue sections were counterstained with Harris hematoxylin (BBC Biochemical). Immunofluorescence staining was performed by fixing the frozen sections with 4% PFA. The sections were then permeabilized with 0.2% Tween-20 in PBS, blocked in 5% horse serum, stained with primary antibodies, and incubated with goat antirat Alexa 568 and goat antirabbit 488 antibodies (Thermo Fisher Scientific). Nuclei were stained with DAPI. Images were taken with a DP72 digital camera mounted onto a BX51 microscope (Olympus Corp).

### Statistical analysis

Data are presented as the means ± SEMs. All data were analyzed using the GraphPad Prism 4.5 software package (GraphPad Software). A *P*-value less than 0.05 was considered statistically significant. Each experiment was repeated three or more times, and reproducible results were obtained. Representative data are shown in the figures.

## Results

### Peli1 is aberrantly upregulated in psoriatic plaques

**Fig. 1:**
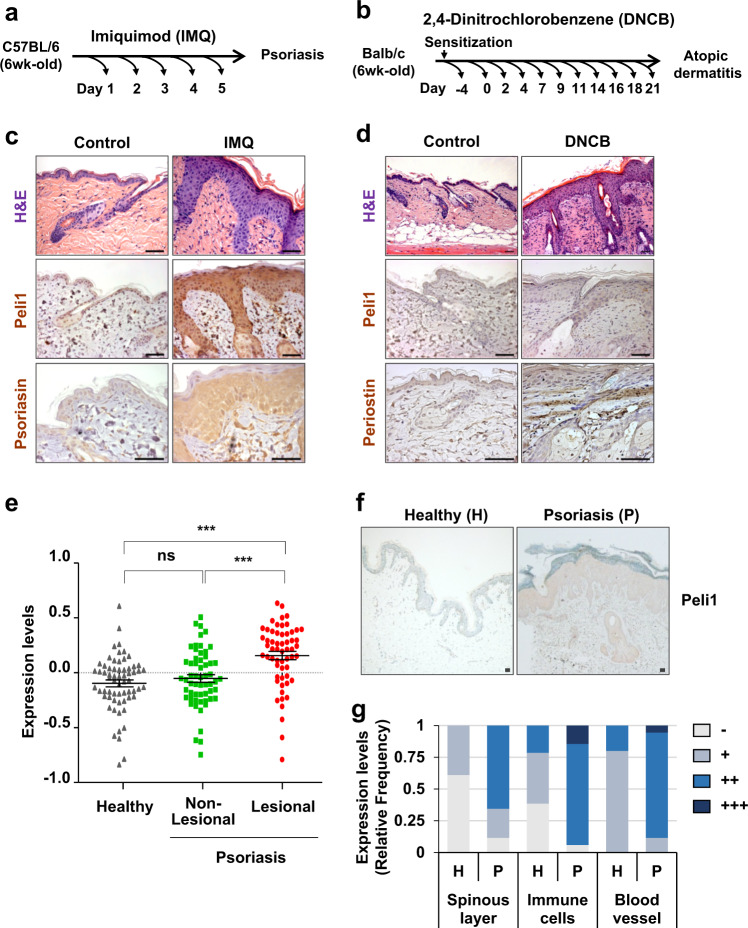
Correlation between Peli1 expression and the development of psoriasis-like skin inflammatory disease. **a** Psoriasis-like skin inflammation was induced by topical application of imiquimod (IMQ) cream daily for six consecutive days. Control (Cont) indicates equivalent volume of Vaseline treatment. **b** To induce atopic dermatitis-like symptoms, dinitrochlorobenzene (DNCB) was topically applied to BALB/c mice. One day after dorsal hair removal, 1% DNCB was applied. Five days after dorsal hair removal, 0.2% DNCB was applied as described in the “Materials and methods” section. **c** Psoriasis-like skin generated by IMQ cream. H&E-stained sections of skin (top) and immunohistochemistry of Peli1 (middle) and Psoriasin (bottom) encoded by the S100A7 gene overexpressed in psoriasis. Scale bars, 50 μm. **d** Atopic dermatitis-like skin generated by DNCB application. H&E-stained sections of skin (top) and immunohistochemistry of Peli1 (middle) and Periostin (bottom) encoded by the S100A7 gene with overexpression in psoriasis. Scale bars, 50 μm. **e** Comparison of Peli1 mRNA levels in healthy human skin (*n* = 64) and nonlesional skin (*n* = 58) and lesional skin (*n* = 58) samples of psoriasis patients. Microarray data sets processed with the robust multichip average (RMA) method were retrieved from the Gene Expression Omnibus (GEO) Database (accession number: GSE 13355). Expression values in the dot graph are adjusted for RMA expression values (log scale) to account for batch and sex effects. Data are presented as the means ± SEMs. *P < 0.05, **P < 0.01, ***P < 0.001; ns not significant based on Student’s *t*-test. **f** Normal human skin (breast) immunostained for Peli1 (left 40×). Psoriasis lesion immunostained for Peli1 (right, 40×). Scale bars, 50 μm. **g** Peli1 expression was evaluated semiquantitatively by comparing the intensity of expression between healthy skin (H) and psoriatic skin lesions (P) in the spinous layer and infiltrated immune cells.

Treatment with a TLR agonist triggers a signaling cascade in innate immune cells^21^. Inappropriate TLR activation has been linked to the pathogenesis of autoimmune diseases, including psoriasis^22,23^. Topical application of IMQ, an effective agonist of TLR7 in mice (TLR7 and TLR8 in humans), can induce and exacerbate psoriasis by activating the IL-23/Th17 pathway via TLR7^24,25^. Interestingly, we found that stimulation with an agonist of TLR3, TLR4, TLR5, or TLR6 induced a significant increase in the expression of Peli1 protein (Supplementary Fig. 1). We found that Peli1 expression was induced after stimulation with topical IMQ treatment (Fig. 1a, c). Hematoxylin and eosin-stained skin sections derived from IMQ-treated mice revealed psoriasiform dermatitis with features of a thickened cornified layer (hyperkeratosis), epidermal hyperplasia (acanthosis), and regular elongation of the rete ridge (Fig. 1a, c, and data not shown). Interestingly, the expression of Peli1 was significantly increased in IMQ-treated psoriatic skin lesions but not in control-treated healthy skin. Peli1 expression was characterized by moderate or strong cytoplasmic/nuclear positive staining in basal and spinous layers. In IMQ-induced psoriatic dermis, fibroblasts and infiltrated immune cells in skin lesions also showed strong nuclear staining of Peli1 (Fig. 1c).

Next, we tested Peli1 expression in lesional specimens obtained from 2,4-dinitrochlorobenzene (DNCB)-induced atopic dermatitis, a model of inflammation (Fig. 1b, d). Generally, topically applied DNCB can complex with various skin proteins to form covalent conjugates that act as immunogens. DNCB complex proteins are internalized by skin Langerhans cells, dermal dendritic cells, and macrophages. They are then processed and presented to T cells for activation^26^. Psoriasis and atopic dermatitis are similar in that they are characterized by altered growth and differentiation of epidermal keratinocytes. However, psoriasis and atopic dermatitis display different T cell polarities and different arrays of cytokines. Psoriasis is a disease driven by Th17 cells associated with IL-17 activation, while atopic dermatitis has a strong Th2 component associated with IL-4 and IL-13 overproduction^27^. Although the treatment of mouse skin with DNCB increased the thickness of the epidermis and recruitment of immune cells to the dermis along with production of secretory Periostin, a matricellular protein known to contribute to clinical features of atopic dermatitis^28,29^, we failed to detect the induction of Peli1 in atopic dermatitis-like inflammation (Fig. 1d).

To investigate the expression profile of Peli1 protein in chronic inflammatory skin diseases, Peli1 expression was characterized by comparing nonlesional and lesional skin of psoriasis patients initially via RNA profiling using data retrieved from open database analytics^30^. The expression of Peli1 was barely detectable in most healthy human skin samples (Fig. 1e), while it was highly upregulated in lesional skin samples derived from psoriasis patients. However, the expression levels of Peli1 in nonlesional skin samples were similar to those in healthy skin samples (Fig. 1e). To further analyze the Peli1 protein expression profile in chronic inflammatory skin diseases, we collected skin samples from psoriasis patients and heathy controls and compared the Peli1 levels. In healthy skin samples, Peli1 was mainly expressed in the cytoplasm of epidermal keratinocytes of the basal layer, although it was also expressed in a few dermal endothelial cells and immune cells (Fig. 1f). However, in skin plaques and lesions obtained from psoriasis patients, Peli1 expression was upregulated in the cytoplasm and a few nuclei of the entire layer of epidermal keratinocytes except the stratum corneum (Fig. 1g). In infiltrated immune cells, Peli1 was expressed at moderate-to-high intensities. It was also expressed in endothelial cells (Fig. 1g). A strong upregulation of Peli1 expression was observed in the entire epidermis, including in infiltrated immune cells and blood vessels of skin lesions derived from psoriasis patients compared with those derived from healthy skin (Fig. 1g). These results indicate that Peli1 expression might be highly correlated with the development of psoriasis.

### Activation of Peli1 expression causes epidermal hyperplasia and psoriasis-like skin lesions

**Fig. 2:**
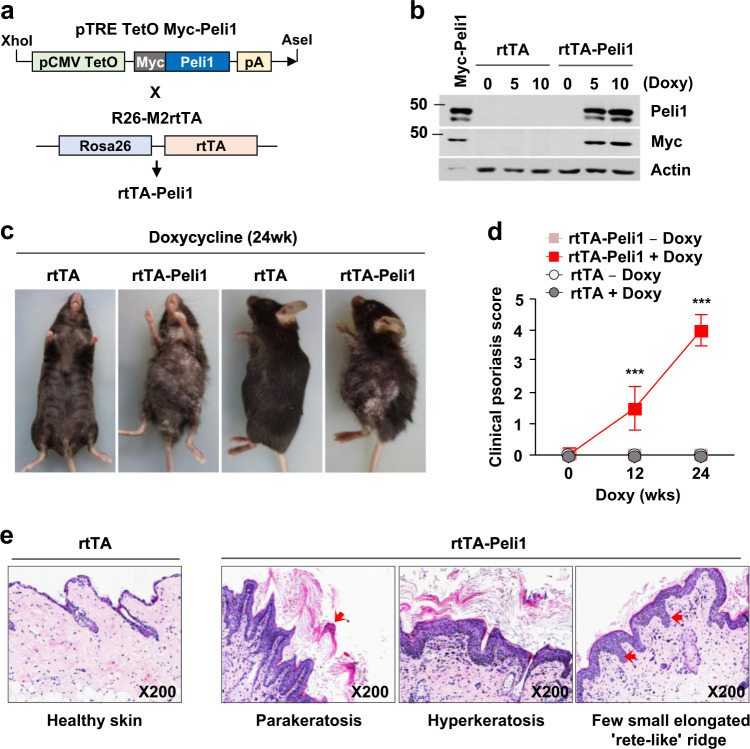
Development of epidermal hyperplasia and psoriasis-like skin inflammation in inducible transgenic mice expressing the human Peli1 gene. **a** Generation of inducible human Peli1 transgenic mice. The cDNA sequence for human Peli1 was placed under the Tet-responsive promoter and introduced into fertilized mouse oocytes. To generate rtTA-Peli1 mice, the founders were crossed with R26-M2rtTA mice (B6.Cg-Gt(ROSA)26Sor^tm1(rtTA*M2)Jae^/J). For inducibility, Myc epitope-tagged human Peli1 gene sequences under the control of the TetO promoter and human early cytomegalovirus enhancer were included. **b** Immunoblotting analysis of tissues derived from rtTA and rtTA-Peli1 mice using antiPeli1 and antiMyc antibodies revealing Peli1 protein expression. Doxy doxycycline treatment. **c** Adult rtTA control mice and rtTA-Peli1 inducible transgenic mice were treated with or without doxycycline (2 mg/mL) for 12 weeks. Offspring of three rtTA-Peli1 founder lines exhibited macroscopic pathologies after doxycycline administration. More than 95% of rtTA-Peli1 mice showed several psoriasis-like features, including extensive erythema, hair loss, severe pruritus, and loosely adherent silver-white scaling. **d** Clinical psoriasis scores of rtTA and rtTA-Peli1 mice treated with or without doxycycline are shown. Scores derived from 3 to 5 mice per group. Data are presented as the means ± SEMs. ***P < 0.001, two-way ANOVA. **e** H&E staining and immunostaining of representative skin sections.

Since Peli1 was highly upregulated in psoriatic skin lesions, we generated double-positive mice carrying both the human Peli1 transgene and rtTA inducer allele (Peli1+/ROSA26-rtTA+ doxycycline inducible Peli1 transgenic mice, hereafter referred to as rtTA-Peli1) (Fig. 2a and Supplementary Fig. 2). Immunoblotting analysis showed detectable levels of human Peli1 protein only in double-transgenic rtTA-Peli1 mice after doxycycline treatment (Fig. 2b and Supplementary Fig. 2). However, nontransgenic rtTA mice treated with doxycycline did not express any human Peli1 (Fig. 2b). Doxycycline-inducible rtTA-Peli1 mice showed no detectable human Peli1 protein when they were untreated (Fig. 2b). Offspring of three rtTA-Peli1 founder lines exhibited macroscopic pathologies as early as 10–12 weeks after doxycycline administration (Fig. 2c, d). By week 12 postdoxycycline treatment, more than 95% of rtTA-Peli1 mice showed several psoriasis-like features, including extensive erythema, hair loss, severe pruritus, and loosely adherent silver-white scaling (data not shown, Fig. 2d, e). Histological analysis of skin samples derived from treated 4-month-old rtTA-Peli1 mice revealed acanthosis, regularly elongated rete ridges, a thickened cornified layer (hyperkeratosis), epidermal hyperplasia (acanthosis), and parakeratosis (Fig. 2e). The severity of symptoms based on PASI scores correlated with the duration of Peli1 overexpression (Fig. 2d). However, rtTA mice treated with doxycycline or rtTA-Peli1 mice without doxycycline treatment did not show any sign of skin inflammation (Fig. 2d).

### Overexpression of Peli1 in epidermal cells initiates the development of psoriasis-like disease

**Fig. 3:**
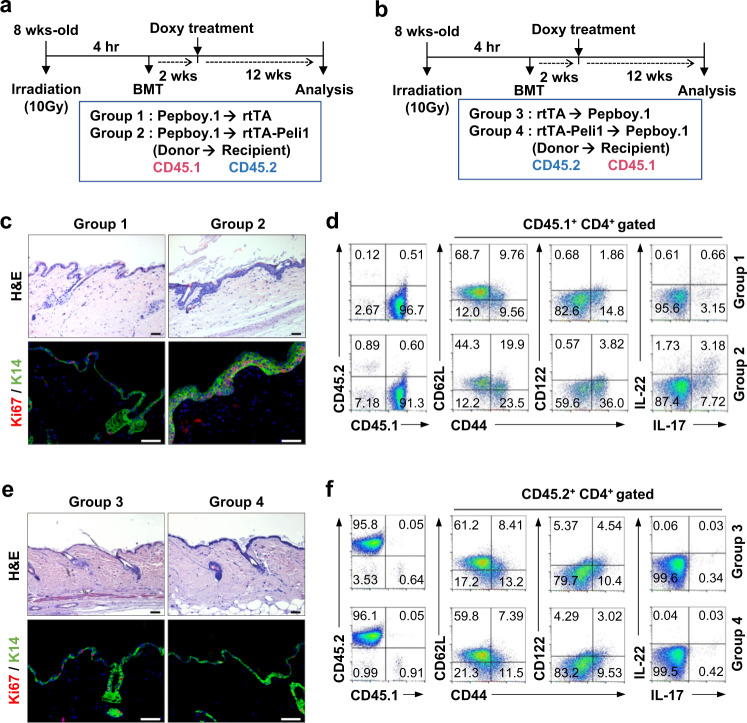
Overexpression of Peli1 in epidermal cells but not in T cells triggers the development of psoriasis-like disease. **a**, **b** Schematic representation of the generation of chimeric recipient mice. Bone marrow (BM) cells were isolated from donor mice and injected into lethally irradiated recipient mice. The following four groups were generated: Pepboy.1 (CD45.1) BM →rtTA (CD45.2) mice (group 1); Pepboy.1 (CD45.1) BM → rtTA-Peli1 (CD45.2) mice (group 2); rtTA (CD45.2) BM → Pepboy.1 (CD45.1) mice (group 3); and rtTA-Peli1 (CD45.2) BM → Pepboy.1 (CD45.1) mice (group 4). After BMT, each group of mice was treated with doxycycline for 12 weeks and then sacrificed (*n* = 3 for each group). **c**, **e** H&E-stained sections of skin from each group of mice treated with doxycycline for 12 weeks. Scale bar, 50 μm. Immunofluorescence of keratin 14 and Ki67 expression. Scale bar, 50 μm. **d**, **f** Representative FACS plots showing the efficiency of bone marrow transplantation, activation of T cells, and intracellular expression of IL-17 and IL-22. Gating blood cells for CD45.1 and CD45.2 expression revealed more than 90% engraftment of donor cells in recipient mice. Lymphocytes were isolated from draining lymph nodes and stained for CD45.1, CD45.2, CD4, CD44, CD62L, CD122, IL-17, and IL-22, followed by flow cytometry.

To dissect the pathways by which Peli1 expression induces the development of psoriasis, we utilized the adoptive transfer function of congenic mouse bone marrow (BM) cells. BM cells were isolated from donor mice and injected intravenously into lethally irradiated recipient rtTA and rtTA-Peli1 mice (Fig. 3a) or Pepboy.1 mice (Fig. 3b). After BM transfer, each group was analyzed for psoriasis-like phenotypes at 12 weeks postdoxycycline treatment. Interestingly, only rtTA-Peli1 chimeric mice receiving Pepboy.1 BM cells (group 2) developed psoriatic-like lesions, including epidermal thickening, scaling, small rete ridges, and abnormal proliferative basal keratinocytes of the epidermis (Fig. 3c). We also found a significant abnormality in the ratio of naïve-to-activated T cells in rtTA-Peli1 mice receiving Pepboy.1 BM cells (group 2) (Fig. 3d). To determine the percentages of Th1-positive, Th2-positive, and Th17-positive cells in the cutaneous lymph nodes, we also analyzed the expression profile of intracellular IFNγ, IL-4, IL-17a, and IL-22 by flow cytometry. Of interest, rtTA-Peli1 mice receiving Pepboy.1 BM cells (group 2) showed an increased percentage of IL-17^+^IL-22^+^CD4^+^ T cells compared with that in the mice in the other experimental groups (Fig. 3d). Further histopathological analysis showed that Pepboy.1 chimeric mice receiving rtTA-Peli1 BM cells (group 4) showed no significant signs of psoriasis-like phenotype (Fig. 3e, f), implying that overexpression of Peli1 in lymphocytes only was insufficient to develop psoriatic lesions. To assess the effect of Peli1 overexpression on T cell activation, we stimulated rtTA and rtTA-Peli1 CD4^+^ T cells with antiCD3 and antiCD28 antibodies in vitro. Our results showed that T cells were hyperactivated in doxycycline-treated rtTA-Peli1 mice, whereas overexpression of Peli1 in T cells had no significant effect on the activation of TCR signaling after TCR stimulation (Supplementary Fig. 3a). To further investigate the differentiation capacity of Peli1-overexpressing T cells, we performed in vitro polarization using MACS-purified CD4^+^ T cells differentiated into Th1, Th2, and Th17 cells. Our results showed that the polarization potentials of Peli1-overexpressing T cells toward their respective Th1 (driven by IL-12 and antiIL-4), Th2 (driven by IL-4 and antiIFNγ), and Th17 (driven by TGFβ, IL-6, antiIFNγ, and antiIL-4) lineages were similar to those of control T cells (Supplementary Fig. 3b). Taken together, these data indicate that Peli1-mediated hyperactivation of keratinocytes can induce pathogenic hyperactivation of T cells in the pathogenesis of skin inflammation and psoriasis lesions. Peli1 activation in T cells is insufficient to trigger psoriasis-like disease, while T cells are indispensable for establishing the disease.

### Activation of Peli1 expression triggers epidermal hyperplasia and a subsequent Th17 cell response

**Fig. 4:**
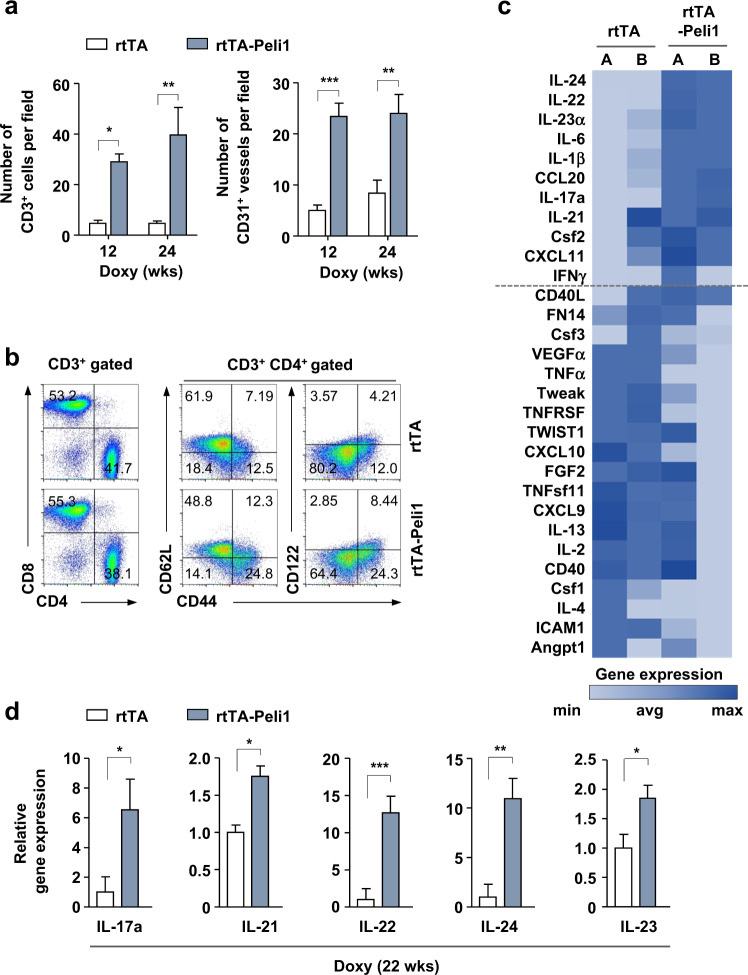
Peli1 induces the development of psoriasis-like disease via a Th17 cell response. **a** Adult rtTA control mice and rtTA-Peli1 inducible transgenic mice were treated with doxycycline (2 mg/mL) for 12 and 24 weeks. Quantification of CD3^+^ cells and CD31^+^ vessels based on five skin sections obtained from five independent mice with the indicated genotypes (*n* = 3 mice per genotype). **b** Flow cytometry of T cells derived from rtTA and rtTA-Peli1 mice following treatment with doxycycline for 24 weeks. Immunocytes were isolated from the draining lymph nodes (inguinal, axillary, brachial, and cervical lymph nodes) of rtTA and rtTA-Peli1 mice. Cells were stained with CD3, CD4, CD44, CD62L, and CD122, followed by flow cytometry. Gated CD3^+^CD4^+^ T cells were analyzed for CD62L, CD44, and CD122 expression. **c** Heat map depicting real-time quantitative PCR (qRT-PCR) analysis of inflammation-related genes in skin samples obtained from doxycycline-treated rtTA and rtTA-Peli1 mice (two representing each genotype). Genes were ranked based on fold change in expression. The expression of genes above the dashed line was highly elevated in lesional skin samples derived from mice overexpressing Peli1 (rtTA-Peli1 mice with doxycycline treatment). **d** Comparison of Th17-related cytokine levels in doxycycline-treated rtTA and rtTA-Peli1 mice. Quantitative RT-PCR for cytokine expression was performed using skin tissues. Data are presented as the means ± SEMs (*n* = 3). *P < 0.05, **P < 0.01, ***P < 0.001; ns not significant; two-way ANOVA.

Increased epidermal infiltrations of CD3^+^ T cells and F4/80^+^ macrophages and expansion of CD31^+^ endothelial cells were observed in the skin tissue of rtTA-Peli1 mice compared with those of in the skin tissue of rtTA mice treated with doxycycline (Fig. 4a). Immunohistochemical analysis of rtTA-Peli1 showed high similarity with phenotypes of human psoriatic skin (Table 1)^31,32^. Psoriatic lesions are characterized by the infiltration of T cells that secrete IFNγ, TNFα, IL-17, and IL-22^33^. Since the cutaneous lymph nodes of doxycycline-treated rtTA-Peli1 mice were enlarged with elevated levels of cellularity (data not shown), we assessed the activation status of CD4^+^ T cells based on the surface expression of their activation markers CD44, CD62L, and CD122 (Fig. 4b). Doxycycline-treated rtTA mice did not show obvious abnormalities in the frequency of naïve or activated/memory T cells (Fig. 4b). However, doxycycline-treated rtTA-Peli1 mice showed an increased frequency of activated/memory T cells, with reduced naïve T cells in cutaneous lymph nodes (Fig. 4b). Furthermore, a higher number of IL-17^+^ cells and IL-22^+^ CD4^+^ T cells were detected in cutaneous lymph nodes of rtTA-Peli1 mice treated with doxycycline than in their counterparts (Supplementary Fig. 4a). Considering that IL-17-producing γδ T cells also play a critical role in the pathogenesis of psoriasis, we analyzed IL-17 production in γδ T cells in draining lymph nodes using flow cytometry. We found that IL-17 production by γδ T cells in rtTA-Peli1 and rtTA mice showed a similar pattern (Supplementary Fig. 4b).

**Table 1:** Comparison of pathological phenotype similarity between human psoriasis and Peli1 expressing mice.

	Human psoriasis	Phenotype in Peli1 expressing mice
Epidermal changes		
Thickening	Y	Y
Altered differentiation	Y	Y
Rete ridges	Y	Y
Papillomatosis	N	N
Vascular changes		
Dilation of capillary loops	Y	Y
Inflammatory changes		
Epidermal T cell infilteration	Y	Y
Intra-epidermal microabscesses	Y	Y
others		
“Koebner” phenomenon	Y	nd
Phenotype dependent on immune activation	Y	Y
Features unlike human psoriasis		Retarded growth, hair abnormality

*Y* yes, *N* no, *nd* not yet determined.

Psoriatic plaques are formed due to dysregulated interactions between innate and adaptive components of the immune system affecting resident immune cells of the skin^32^. To gain insight into the molecular pathogenesis of Peli1-induced psoriasis, we analyzed a panel of key inflammation-related genes usually activated during psoriasis using an RT^2^ profiler PCR array (Fig. 4c). We found significant changes in the expression levels of several Th17-related cytokines, such as those secreted by Th17 cells (IL-17a, IL-21, IL-22, and IL-24), cytokines involved in Th17 differentiation (IL-21 and IL-23), and the chemokine CCL20 involved in the trafficking of Th17 cells. Skin samples of rtTA-Peli1 mice showed strong upregulation of the levels of different cytokines, IL-17a (6.5-fold), IL-21 (1.7-fold), IL-22 (12.5-fold), IL-24 (10.9-fold), IL-23 (1.8-fold), and CCL20 (1.7-fold), compared with those in skin samples of rtTA mice (Fig. 4d). Taken together, our results strongly suggest that increased Peli1 expression triggers the development of chronic skin inflammation, similar to the phenotype of human psoriatic skin (Table 1)^31,32^.

### S and G2/M cell cycle activation of epidermal keratinocytes by Peli1 expression

**Fig. 5:**
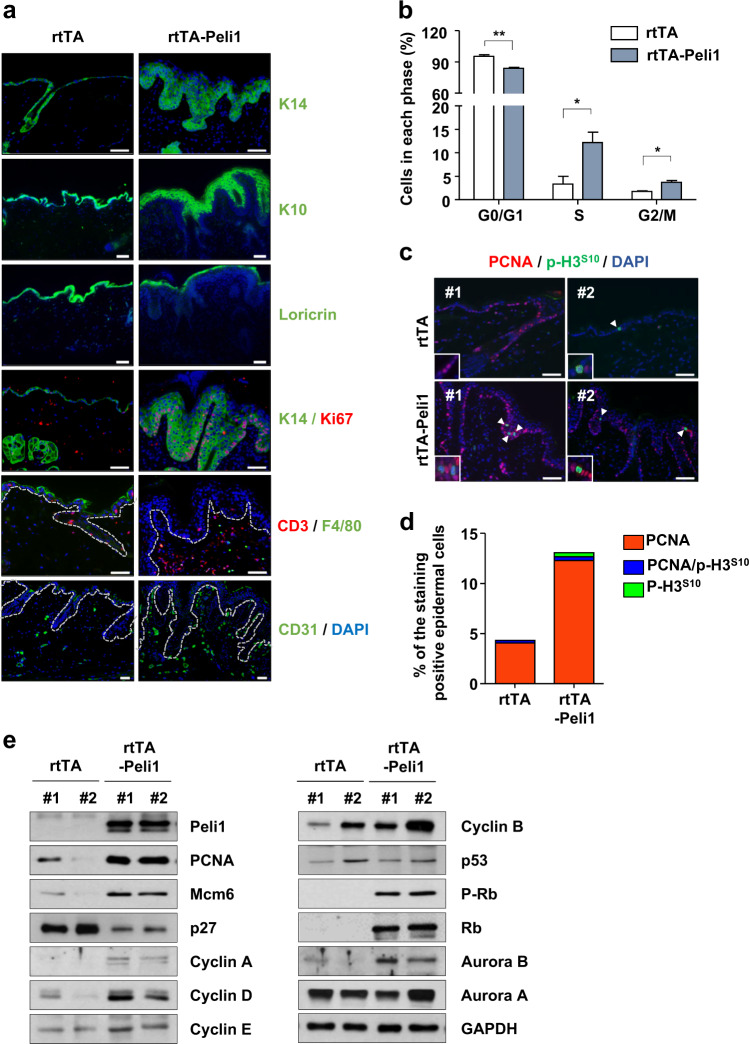
Activation of Peli1 expression causes epidermal hyperplasia and leads to aberrant cell cycle progression at S-G2/M phases. **a** Adult rtTA control mice and rtTA-Peli1 inducible transgenic mice were treated with doxycycline (2 mg/mL) for 12 weeks. Representative immunofluorescence images of skin sections showing the expression of keratinocyte differentiation markers (keratin 14, keratin 10, and loricrin), a proliferation marker (Ki67), dermal infiltrated immune cell markers (CD3 and F4/80), and an angiogenesis marker (CD31). The dotted line indicates the border between the epidermis and dermis. Scale bars, 50 μm. **b** Primary keratinocytes were isolated from rtTA or rtTA-Peli1 mice after 24 weeks of doxycycline treatment. Single-cell suspensions of primary keratinocytes were fixed and stained with propidium iodide for DNA analysis via flow cytometry. Summary data are shown. Data are presented as the means ± SEMs (*n* = 3). *P < 0.05, ** *P* < 0.01, two-way ANOVA. **c** Double immunofluorescence staining for PCNA (red) and phospho-H3^S10^ (green) expression in skin sections of rtTA and rtTA-Peli1 mice after 24 weeks of doxycycline treatment. Scale bars, 50 μm (inset magnification, ×630). **d** Quantification of positively stained keratinocytes in skin sections. PCNA: a marker of S phase; phospho-H3^S10^: a marker of mitosis; PCNA and phospho-H3^S10^ double-positive: a marker of G2 phase. The results are presented as the means ± SEMs (*n* = 5). **e** Immunoblot analysis of the indicated protein in skin tissues derived from rtTA and rtTA-Peli1 mice after 24 weeks of doxycycline treatment.

Epidermal keratinocytes play a key role in innate immunity by inducing and switching classes of T cells recruited to lesional skin^1,3,34^. Therefore, we investigated the molecular mechanism underlying the activation of keratinocyte proliferation and the development of psoriasis induced by overexpression of Peli1. Our immunohistochemical staining results showed an increase in the number of keratinocytes expressing K14 (expressed in mitotically active basal epidermal layer cells), K10 (expressed in abnormally differentiated cells in the epidermal layer), loricrin (a terminally differentiating structural protein in the cornified envelope), and Ki67 (strictly associated with cell proliferation) in mice overexpressing human Peli1 protein (Fig. 5a). In addition, increased epidermal infiltration of CD3^+^ T cells and F4/80^+^ macrophages was detected following the expansion of CD31^+^ endothelial cells in rtTA-Peli1 mice compared with those in rtTA mice treated with doxycycline (Fig. 5a). We next compared the cell cycle profiles of rtTA and rtTA-Peli1 epidermal keratinocytes by flow cytometry (Fig. 5b). We also monitored the expression of PCNA (an S phase marker), PCNA and phospho-H3^S10^ dual expression (a G2 phase marker), and the expression of phospho-H3^S10^ (a marker of mitosis) (Fig. 5c, d). The overall cycle progression in rtTA-Peli1 epidermal keratinocytes was significantly accelerated compared with that in rtTA epidermal keratinocytes of the same age, showing significant differences in G0/G1 phase and rapid progression into S, G2, and M phases induced by Peli1 expression.

We next compared the expression profiles of a series of representative markers involved in the signaling pathways of hyperproliferation and hyperactivation of keratinocytes using skin tissues isolated from rtTA and rtTA-Peli1 mice at 24 weeks after doxycycline treatment (Fig. 5e). The rtTA-Peli1 mice treated with doxycycline for 24 weeks promoted severe psoriasis (Fig. 2). Healthy skin (rtTA) and Peli1-induced psoriatic skin lesions (rtTA-Peli1 mice treated with doxycycline for 24 weeks) showed significant differences in several markers of cell cycle progression, including elevated expression of PCNA, Mcm6, cyclins A, B, D, and E, phosphorylation of Rb at serine 807/811 residues, and Aurora B. However, the level of the Cdk inhibitor p27 was reduced, indicating that Peli1 expression induced cell cycle activation of lesional keratinocytes.

We further examined whether cytokines and chemokines mediated the pathogenesis of psoriasis by overexpression of Peli1 in keratinocytes (Supplementary Fig. 5a). We transfected a Peli1 overexpression vector into HaCaT cells, an immortalized keratinocyte cell line, and found that overexpression of Peli1 induced a significant increase in the expression of IL-21 and IL-24. Consistent with the elevated expression of IL-21 and IL-24, we detected increased activation of STAT3 signaling in the skin tissue of rtTA-Peli1 mice (Supplementary Fig. 5b). The results suggest that IL-21 is highly expressed in lesional psoriatic skin^35^ and that IL-24-mediated activation of STAT3 in the epidermis triggers psoriasis-like skin inflammation^36^. The increased levels of IL-21 and IL24-mediated activation of STAT3 in skin epidermal cells serve as a pathogenic signal and trigger psoriatic skin inflammation in rtTA-Peli1 mice.

## Discussion

**Fig. 6:**
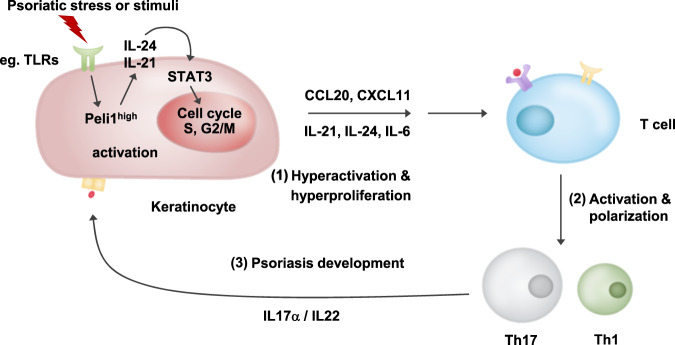
Model of Peli1-mediated cell cycle activation, which generates a feedback cross-link between keratinocytes and the T cell response in the psoriatic microenvironment. A detailed discussion of this model is provided in the text.

Sequential signaling connections between effectors of the innate and adaptive immune systems shape the chronic inflammatory process. Activation of Peli1 appears to be a common mediator in the psoriatic intercellular signaling pathway ranging from keratinocytes to Th17 and Th1 cells. Psoriatic keratinocytes are rich sources of antimicrobial peptides that can trigger the activation of intracellular signaling cascades in T cells^3,34^. Overexpression of Peli1 in keratinocytes induces upregulation of IL-21 and IL-24, activation of STAT3 signaling, and the secretion of mediators underlying the differentiation of Th17 and Th1 cells. These T cells in turn secrete other mediators (e.g., IL-17a and IL-22) and produce proinflammatory cytokines, chemokines, and other components that feed back into the psoriatic inflammatory cycle (Fig. 6).

In this study, adoptive transfer experiments revealed that Pepboy.1 chimeric mice that received Peli1-overexpressing BM cells did not show significant signs of a psoriasis-like phenotype, indicating that overexpression of Peli1 only in T cells was insufficient to trigger the development of psoriasis. Peli1 mediates rhinovirus-induced expression of the CXC-chemokine ligand 8 (CXCL8) chemoattractant in bronchial epithelial cells^37^. In addition, Peli1 is upregulated in neutrophilic asthma patients^19^. These results suggest that Peli1-induced chemokine synthesis might affect the development of airway inflammation in asthma and the respiratory epithelium after viral infection. Interestingly, the results of this study suggest that Peli1-mediated hyperproliferative keratinocytes upregulate the expression and secretion of the CCL20 and CXCL11 chemokines known to be indispensable for chemoattraction. The CCL20 and CXCL11 chemokines can also induce immature DCs and effector/memory T cells in combination with cytokines. Thus, Peli1-mediated hyperactivation of keratinocytes triggers pathogenic hyperactivation of T cells and subsequent cross-talk between keratinocytes and T cells during the pathogenesis of skin inflammation and psoriasis lesions. This study further suggests that selective inhibition of T cell activation or polarization alone may be insufficient to cure psoriasis. Thus, inhibition of both keratinocyte hyperactivation and polarized Th17 and Th1 cell responses might be a better strategy for psoriasis treatment. Recently developed biologic agents can also be used to selectively target the IL-23–Th17 cell axis, including the cytokines IL-23 and IL-17 and their receptors and IL-22, for treating psoriasis^2^. Although TNFα-targeting and IL-17a-targeting drugs have been found to be effective, a targeting strategy that inhibits Peli1 might be a more powerful approach to treat psoriasis. Indeed, our unpublished observation showed that systemic delivery of cyclosporine A or methotrexate, which are effective and widely used medications for treating psoriasis and other inflammatory diseases, showed significantly improved antipsoriatic effects in a Peli1-induced psoriasis mouse model, indicating that Peli1 might be a target for treating psoriasis.

Studies investigating the pathogenesis of psoriasis have been hindered by the lack of appropriate animal models. IMQ treatment not only induced phenotypic changes consistent with human psoriasis but also showed a dependence on IL-23 and IL-17. However, IMQ-induced psoriasis mouse is an acute model of inflammation without exhibiting a chronic or complex phenotype associated with psoriatic disease. For example, it is not associated with comorbidities, such as arthritis, frequently seen in some human cases of psoriasis^3^. Another comorbidity associated with psoriasis includes B cell lymphoma^38^. Our recent study strongly indicates that Peli1 expression is upregulated in patients with B cell lymphoma and that such upregulation is associated with poor prognosis^13^. Mice overexpressing Peli1 develop a wide range of lymphoid tumors, particularly B cell lymphoma^13^. In the present study, we found that inducible overexpression of Peli1 for less than 6 months triggered severe psoriasis and a few comorbidities but not B cell lymphoma. However, we cannot exclude the possibility that inducible Tg mice might develop B cell lymphoma at a later period, as shown in conventional Peli1 Tg mice overexpressing constitutively low levels of Peli1^13^.

In summary, we identified a novel immunopathogenic mechanism underlying Peli1-mediated chronic skin inflammation mediated by a feedback link between keratinocyte hyperactivation and Th17 cell responses. Our results suggest that inhibition of Peli1 represents a potential therapeutic intervention that is effective against chronic skin inflammation.

## Supplementary information

Supplementary information
