# Supplementary material for: Pellino1 promotes chronic inflammatory skin disease via keratinocyte hyperproliferation and induction of the T helper 17 response
Source: Exp Mol Med. 2020 Sep 1;52(9):1537–49. doi: 10.1038/s12276-020-00489-4 (PMC8080721; doi:10.1038/s12276-020-00489-4)
Supplement: Supplementary file 1 — Supplementary information [file 12276_2020_489_MOESM1_ESM.pdf]

SUPPLEMENTARY INFORMATION

Supplementary Fig 1. Upregulation of Peli1 in response to toll-like receptor signaling.

a, b Immunoblots of bone marrow derived macrophages (BMDM) stimulated with various TLR agonist for indicated times. Sources of chemicals and reagents were as follows: mouse TLR1-9 agonist kit, InvivoGen; PMA (phorbol 12-myristate 13-acetate) and ionomycin, Sigma; MG132 and cycloheximide, A.G Scientific.

Supplementary Fig 2. Generation of doxycycline-inducible transgenic and knock out mice targeting Peli1 gene.

Generation of doxycycline-inducible human Peli1 transgenic mice. cDNA sequence for human Peli1 was placed under Tet-responsive promoter and introduced into fertilized mouse oocytes. To generate rtTA-Peli1 mice, founders were crossed with R26-M2rtTA mice (B6.Cg-Gt(ROSA)26Sor$^{tm1(rtTA*M2)Jae}$/J). For inducibility, Myc epitope-tagged human Peli1 gene sequence under the control of TetO promoter and human early cytomegalovirus enhancer were included. In the absence of doxycycline, rtTA will not recognize TetO sequence. No expression of Peli1 will occur. Addition of doxycycline results in binding of rtTA to the TetO sequence and transcriptional activation of Peli1. Such inducible expression is reversible as withdrawal of doxycycline terminates expression. Immunoblotting of tissues from rtTA and rtTA-Peli1 mice using Peli1 and Myc antibodies revealing robust Peli1 protein expression in most types of tissue.

Supplementary Fig 3. Overexpression of Peli1 in CD4 T cells has marginal effect on

TCR signaling and polarization potential.

a Immunoblotting of cell lysates isolated from rtTA or rtTA-Peli1 $CD4^+$ T cells stimulated by cross-linking with anti-CD3 (2 μg/ml) plus anti-CD28 (2 μg/ml) for the time period indicated. 'p-' indicates phosphorylated form of each polypeptide. **b** *In vitro* polarization assay of MACS-purified $CD4^+$ T cells activated under Th1, Th2, and Th17 conditions. MACS-purified $CD4^+$ T cells from rtTA or rtTA-Peli1 lymph-nodes were differentiated with Th1, Th2, and Th17 conditions for five days and then stimulated with PMA/ionomycin in the presence of brefeldin A before intracellular staining with fluorochrome-conjugated antibodies specific for IFNγ, IL-4, IL-17, and IL-22.

Supplementary Fig 4. IL17 production was increased in T cells but not in gamma delta T cells.

a Representative FACS plots showing intracellular expression of IL-17 and IL-22. Intracellular cytokine staining was performed on lymphocytes collected from draining lymph-nodes of rtTA and rtTA-Peli1 mice after 24 weeks of doxycycline treatment. Lymphocytes were isolated from draining lymph-nodes, stained for CD4, CD3, γδTCR, IL-17, and IL-22, and subjected to flow cytometry analysis.

Supplementary Fig 5. Peli1-overexpressed keratinocytes could secreted psoriasis related cytokines.

a Comparison of cytokine and chemokine in myc (control) and myc-Peli1 (overexpression of Peli1) transfected HaCaT cell line. **b** Immunoblot analysis of indicated protein in skin tissues from rtTA and rtTA-Peli1 mice after 24 weeks of doxycycline treatment.

Supplementary Fig 1.

a

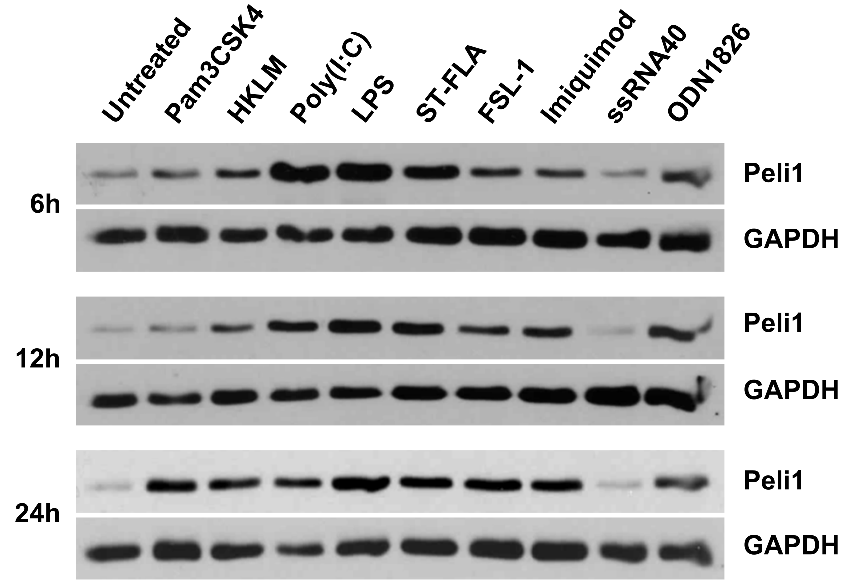

b

Agonist	Target TLRs
Pam3CSK4	TLR1/2
HKLM	TLR2
Poly(I:C)	TLR3
LPS	TLR4
ST-FLA	TLR5
FSL-1	TLR6/2
Imiquimod	TLR7
ssRNA40	TLR7
ODN1826	TLR9

Supplementary Fig 2.

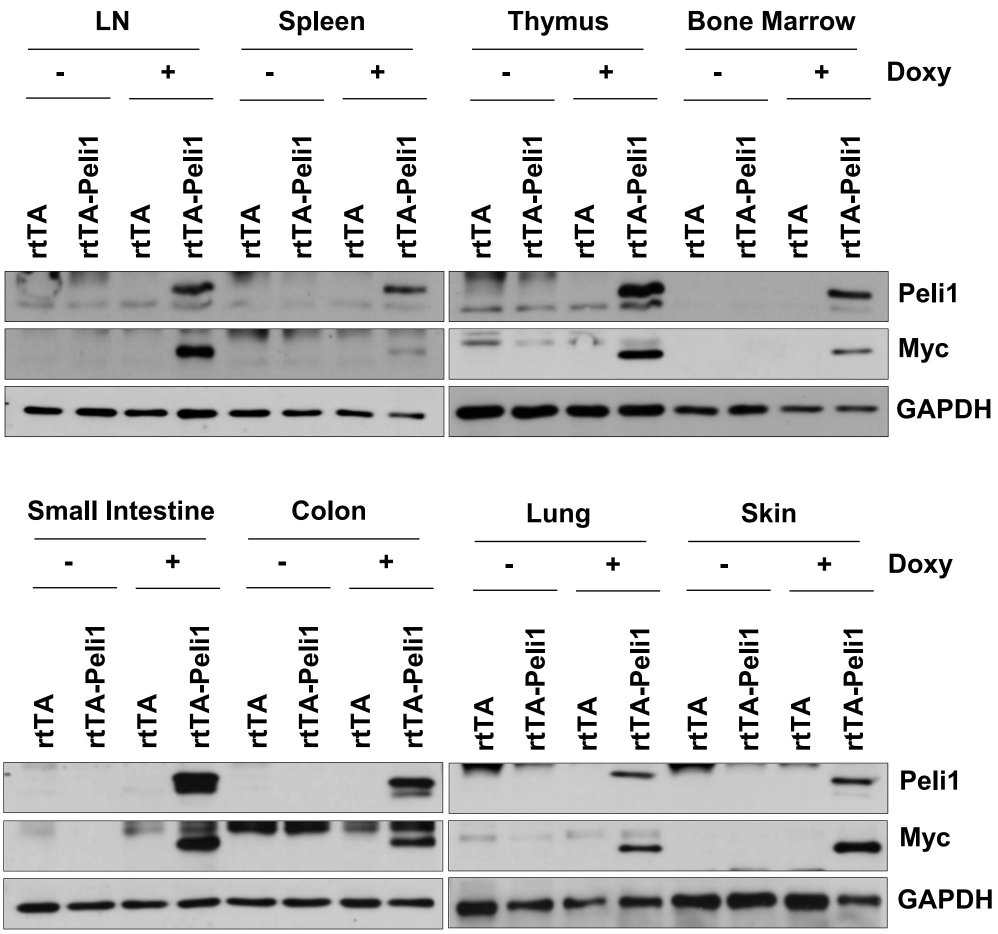

Supplementary Fig 3.

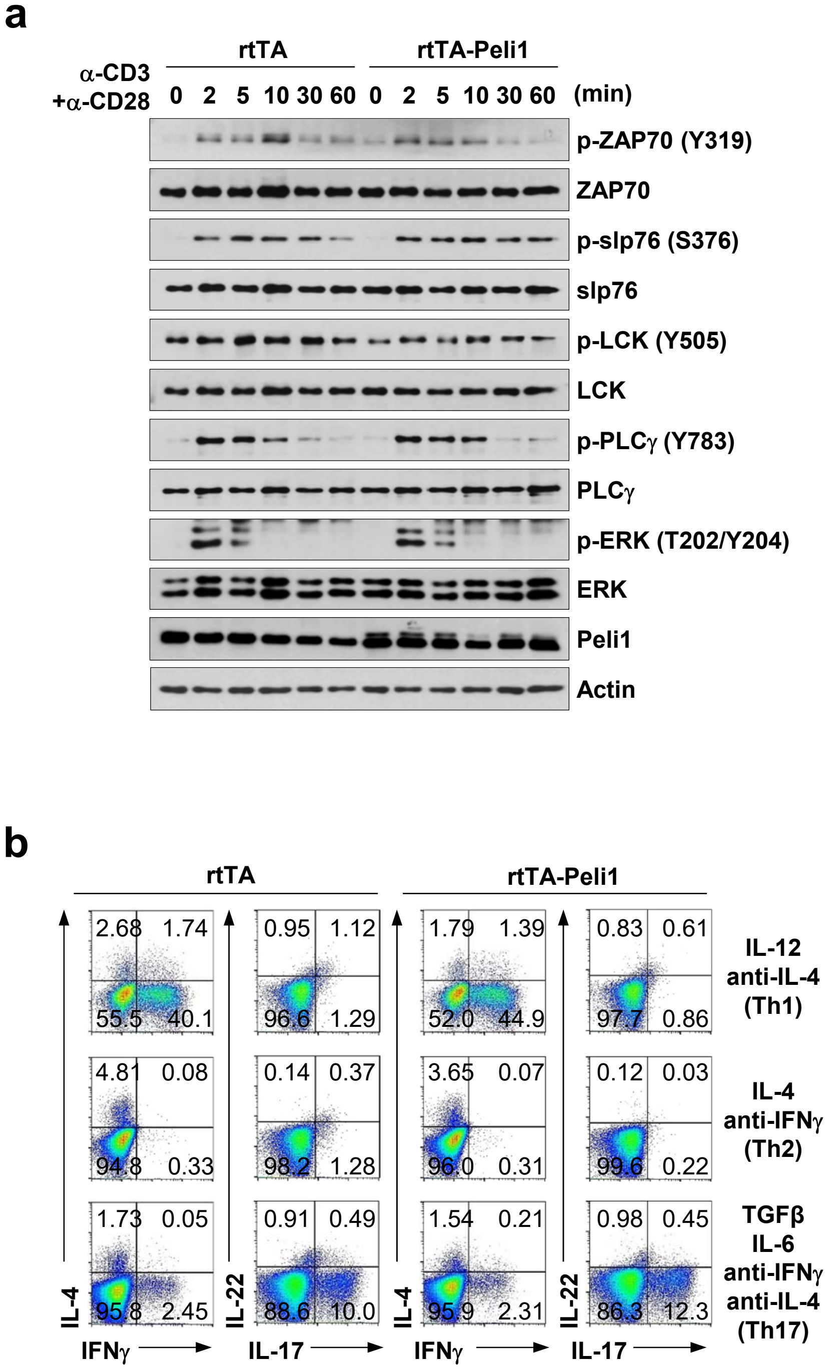

Supplementary Fig 4.

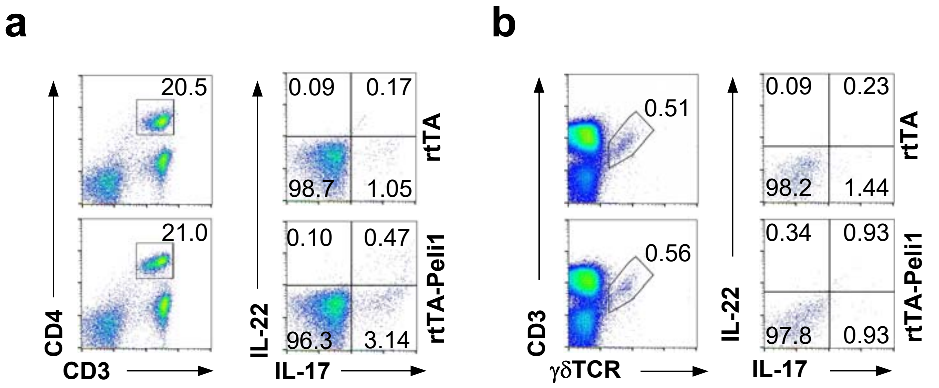

Supplementary Fig 5.

a

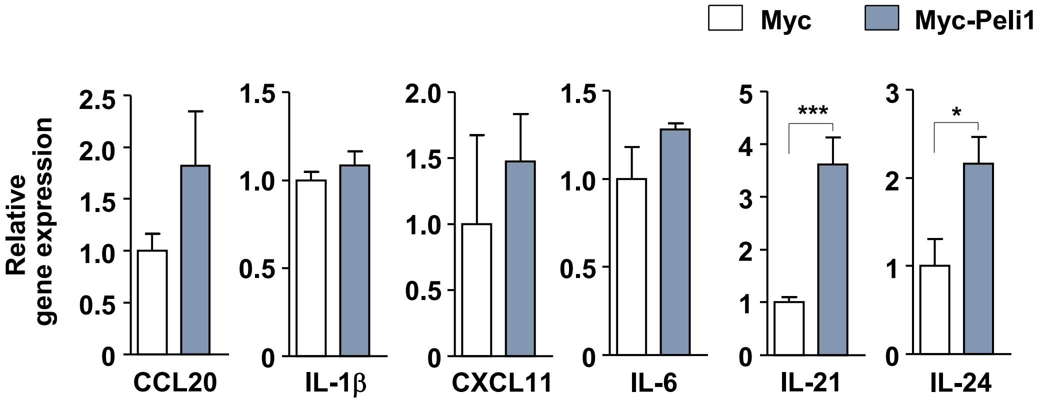

b

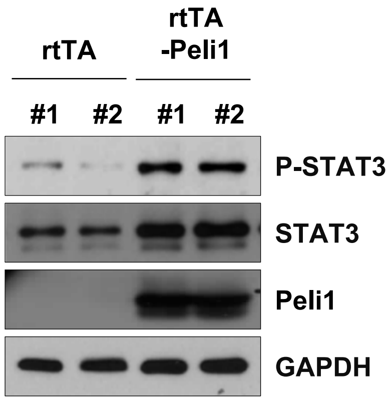

Supplementary Table 1.

Specificity	Clone	Conjugates	Supplier
CD122	TM-b1	APC	eBioscience
CD3e	17A2	Per cy5.5	eBioscience
CD4	RM4-5	PE cy7, APC cy7,	eBioscience
CD44	IM7	FITC, PE cy7	eBioscience
CD45.1	A20	Percp cy5.5, APC cy7	eBioscience
CD45.2	104	PE cy7, PE	eBioscience
CD62L	MEL-14	APC cy7	eBioscience
CD8	53-6.7	PE	eBioscience
IFNγ	XMG1.2	FITC	eBioscience
IL-17	eBio17B7	Percy cy5.5	eBioscience
IL-22	IL22JOP	APC	eBioscience
IL-4	11B11	PE	eBioscience

Supplementary Table 2.

Target	Product #	Target	Product #
Mouse IL-1b	QT01048355	Mouse FN14	QT00255038
Mouse IL-6	QT00098875	Mouse IL-22	QT00128324
Mouse TNFα (TNF)	QT00104006	Mouse IL-23a	QT01663613
Mouse IL-17a	QT00103278	Mouse CD40	QT00155974
Mouse CXCL9	QT00097062	Mouse Tweak (Tnfsf12)	QT00170681
Mouse CXCL10	QT00093436	Mouse ICAM1	QT00155078
Mouse CXCL11	QT00265041	Mouse TWIST1	QT00097223
Mouse CCL20	QT00261898	Mouse Angpt1	QT00166859
Mouse VEGFα	QT00160769	Mouse CD40L (Tnfsf5)	QT00101437
Mouse FGF2	QT00128135	Mouse IL-24	QT01054634
Mouse IFNg	QT01038821	Mouse IL-21	QT00134358
Mouse TNFRSF1α	QT00099547	Mouse Tnfsf11	QT00147385
Mouse IL-2	QT00112315	Mouse IL-4	QT00160678
Mouse Csf1	QT01164324	Mouse IL-13	QT00099554
Mouse Csf2	QT00251286	Mouse GAPDH	QT01658692
Mouse Csf3	QT00105140	Mouse Rn18s	QT02448075

Supplementary Table 3.

Primary antibodies	Product #	Company	Primary antibodies	Product #	Company
Actin	A2066	Sigma	Loricrin	PRB-145P	COVANCE
Akt	4691S	Cell signaling	MCM6	sc-9843	Santa Cruz
Aurora A	sc-56881	Santa Cruz	Myc	A190-105A	BETHYL
Aurora B	sc-25426	Santa Cruz	MyD88	4283	Cell signaling
BubR1	612503	BD Bioscience	P- Histone H3 (S10)	9701	Cell signaling
CD3	ab5690	Abcam	P27	sc-1641	Santa Cruz
CDK4	12790S	Cell signaling	Pellino 1/2	sc-271065	Santa Cruz
Cyclin A	sc-751	Santa Cruz	P-PKCq (T538)	9377S	Cell signaling
Cyclin B1	sc-752	Santa Cruz	P-PLCg (Y783)	14008P	Cell signaling
Cyclin D1	sc-753	Santa Cruz	P-Rb (S807/811)	8516S	Cell signaling
Cyclin E2	sc-28351	Santa Cruz	P-slp76 (S376)	14745P	Cell signaling
ERK	sc-94	Santa Cruz	Psoriasin	ab218207	abcam
F4/80	MCA497GA	AbD serotec	P-STAT3 (S727)	9134P	Cell signaling
Flag	F7425	Sigma	P-ZAP70 (Y493)	2704P	Cell signaling
GAPDH	sc-25778	Santa Cruz	Rb	sc-50	Santa Cruz
HA	G036	abm	Slp76	4958S	Cell signaling
K10	MMS-159S	COVANCE	TRAF3	sc-949	Santa Cruz
K14	PRB-155P	COVANCE	TRAF6	sc-7221	Santa Cruz
Ki67	14-5698	eBioscience	Ubiquitin	sc-8017	Santa Cruz
